# Characterization of SNARE Cleavage Products Generated by Formulated Botulinum Neurotoxin Type-A Drug Products

**DOI:** 10.3390/toxins2082198

**Published:** 2010-08-19

**Authors:** Terrence Hunt, David Rupp, Gary Shimizu, Karen Tam, Julia Weidler, Jack Xie

**Affiliations:** Allergan, Inc., 2525 Dupont Dr, Irvine, CA 92612, USA; Email: rupp_david@allergan.com (D.R.); shimizu_gary@allergan.com (G.S.); tamkaren@gmail.com (K.T.); weidler_julia@allergan.com (J.W.); xie_jack@allergan.com (J.X.)

**Keywords:** abotulinumtoxinA, botulinum toxin, BOTOX^®^, Dysport^®^, incobotulinumtoxinA, onabotulinumtoxin A, SNAP-25, SNARE, syntaxin, Xeomin^®^

## Abstract

The study evaluated substrate cleavage product(s) generated by three botulinum neurotoxin serotype A (BoNT/A) medicinal drug products utilizing a novel and highly specific, light-chain activity, high-performance liquid chromatography (LCA-HPLC) method. Samples were reacted with a commercially available BoNT/A fluorescent substrate derived from the SNAP-25 sequence. Reaction products were separated by reversed-phase HPLC. The method detected an atypical cleavage pattern by one of the formulated drug products. IncobotulinumtoxinA produced two cleavage fragments rather than the single fragment typically generated by BoNT/A. Identification confirmed the secondary cleavage at a position corresponding to SNAP-25 Arg198–Ala199 (normal BoNT/A cleavage is Gln197–Arg198). Arg198–Ala199 is also the cleavage site for trypsin and serotype C toxin. Normal cleavage was observed for all other BoNT/A drug product samples, as well as 900-kD and 150-kD bulk toxin BoNT/A. The reason for this unexpected secondary cleavage pattern by one formulated BoNT/A drug product is unknown. Possible explanations include a contaminating protease and/or damage to the 150-kD type-A toxin causing nonspecific substrate recognition and subsequent cleavage uncharacteristic of type-A toxin. The BoNT/A drug products were also analyzed via the LCA-HPLC assay using a commercial BoNT/C fluorescent substrate derived from the syntaxin sequence. Cleavage of the serotype C substrate by incobotulinumtoxinA was also confirmed whilst neither of the other drug products cleaved the syntaxin substrate.

## 1. Introduction

Seven serotypes (A to G) of *Clostridium botulinum* are known. Each serotype synthesizes a 150-kD neurotoxin (BoNT) along with a group of nontoxic neurotoxin-associated proteins (NAPs) that form complexes [1]. BoNT is a zinc-endopeptidase that is composed of a 100-kD heavy-chain and a 50-kD light-chain, connected by a disulfide bond. The heavy-chain is responsible for binding (*C*-terminal domain) and translocation (*N*-terminal domain), whereas the light-chain is responsible for proteolytic activity. The light-chain of each serotype specifically cleaves one or more soluble *N*-ethylmaleimide—sensitive factor attachment receptors (SNAREs). The endopeptidase activities of the toxins are unique in that they require the reduction of the disulfide bond between the light and heavy chains in order for enzymatic activity to occur. Each serotype cleaves selectively at a specific peptide bond of a specific substrate. For example, the light-chain of BoNT serotype A (BoNT/A) specifically cleaves SNAP-25_1–206_ at position Gln197–Arg198 [2]. This specific cleavage is an intrinsic property of the serotype. 

Serotype A has been formulated into several drug products for therapeutic treatment of various disorders. The formulated drug products differ considerably in both drug substance and drug product composition (see Table 1). 

**Table 1:** BoNT/A drug product composition.

**Non-proprietary Name**	**Brand Name**	**Labeled Potency (LD_50_)**	**Toxin**	**Excipients**
onabotulinumtoxinA	BOTOX^®^	100 Units	BoNT/A900-kD complex	0.5 mg HSA ^a^ 0.9 mg NaCl
abotulinumtoxinA	Dysport^®^	500 units	BoNT/A300/500-kD ^b^ complex	0.125 mg HSA ^a^ 2.5 mg Lactose
incobotulinumtoxinA	Xeomin^®^	100 Units	BoNT/A150-kD	1 mg HSA ^a^ 4.7 mg Sucrose

^a^ Human serum albumin. ^b^ Ipsen Biopharm Limited reported molecular weight range of the complex.

Recently the drug products have been assigned non-proprietary (USAN) names to avoid confusion due to brand names and to emphasize the non-interchangeability of units. However, physicochemical evaluations that might offer functional insight into differences have thus far been limited.

It is important to note that it is the SNARE cleavage (described above) that ultimately produces the desired therapeutic effect by inducing regional disruption of neuronal stimulation of an organ (e.g., muscle or sweat gland). Due to the potent nature of the toxin, extremely small therapeutic concentrations are sufficient; typically nanograms per finished drug product vial. The inherent difficulties in analyzing nanogram quantities of formulated drug substance have relegated most comparisons to the clinical setting. However, compared to physicochemical techniques, clinical readouts can be insensitive while providing limited mechanistic information. What has been lacking is a highly-sensitive endopeptidase activity assay that is not subject to matrix interference due to excipients and allows simultaneous characterization of cleavage specificity.

Existing endopeptidase assays, such as spectrophotometric assays, are insufficiently sensitive to measure drug product concentrations and are subject to drug product matrix interferences. For example, BoNT/A light-chain activity methods utilizing fluorescence resonance energy transfer (FRET) substrates typically measure a change in fluorescent signal directly from the reaction solution. The total fluorescence of the reaction solution, which includes intrinsic fluorescence due to reaction buffer components and toxin formulation excipients, is measured before and after substrate cleavage. An increase or decrease in fluorescence will result, depending on the FRET pair used. This change in fluorescent signal due to cleavage is non-specific and often small in relation to the background signal, resulting in poor sensitivity. Immunoassays represent another technique employed for measuring endopeptidase activity. These assays rely on the availability of special reagents (*i.e.*, high-affinity antibodies for each cleavage product) to achieve specificity [3] and are incompatible with many commonly used formulation excipients. The result is that most assays currently used are inconvenient, product specific and insensitive, usually requiring many product vials to accomplish a single test that results in only limited information.

The light-chain activity high-performance liquid chromatography (LCA-HPLC) method developed and applied here incorporates a light-chain reaction procedure using a commercially available BoNT/A FRET substrate derived from the SNAP-25 sequence coupled with a reversed-phase (RP)-HPLC analysis procedure to detect and quantitate the fluorescently labeled reaction product(s). The method is unique in that the RP-HPLC analysis eliminates matrix interferences by separating the cleaved substrate products from the non-cleaved substrate, other reaction reagents, and product formulation components, thereby significantly increasing the signal-to-noise ratio. This method provides single-vial sensitivity along with high throughput due to the HPLC automation. Additionally, because the method requires only substrate and no product-specific antibodies or reagents, it provides the opportunity to evaluate and compare drug products; for example assessing the effects of formulation excipients, manufacturing processes, and other parameters on light-chain activity and stability.

Another significant advantage of this procedure is the ability to separate, detect and quantify distinct light-chain cleavage products. While methods exist to measure the light-chain activity of BoNTs [4,5], this is the first method (to our knowledge) to selectively determine, as a routine part of the assay, the actual cleavage product(s) generated by formulated BoNT/A drug products. The adaptable nature of the method also allowed simple substitution of an additional substrate derived from the syntaxin sequence, further extending the range of potential characterization to serotype C characterization. The straightforward substitution of substrates extends the utility as an identification test [6].

## 2. Materials and Methods

The present study compared the light-chain enzymatic activities of three BoNT/A drug products: onabotulinumtoxinA (BOTOX^®^, Allergan, Inc.; Irvine, CA, USA), incobotulinumtoxinA (Xeomin^®^, Merz Pharmaceuticals; Frankfurt, Germany), and abotulinumtoxinA (Dysport^®^, Ipsen Biopharm Limited; Wrexham, UK). All drug products were stored according to label instructions and tested within expiration dating. The light-chain activity and cleavage pattern of these BoNT/A products were assessed using the novel LCA-HPLC method reported here with BoNT/A substrate (SNAPtide^®^520; List Biological Laboratories, Inc.; Campbell, CA, USA). SNAPtide^®^520 sequence: Abz Thr D-Arg Ile Asp Gln Ala Asn Gln Arg Ala Thr Lys Nle Lys Dnp; where Abz = *o*-Aminobenzoic acid (FRET pair fluorophore) and Dnp = 2,4-Dinitrophenyl (FRET pair quencher).

BoNT/A cleavage fragment (designated as SNAPtide^®^529 by List Biological Laboratories, Inc.) was utilized to identify retention time of the fragment.

900-kD BoNT/A toxin complex (supplied by Allergan, Inc.; Irvine, CA, USA) and 150-kD BoNT/A holotoxin (supplied by Metabiologics, Inc.; Madison, WI, USA) were used to characterize BoNT/A unformulated bulk toxin cleavage. 

Commercially available BoNT/C FRET substrate (SYNTAXtide^®^560; List Biological Laboratories, Inc.; Campbell, CA, USA) was utilized to assess type-C cleavage. Because of the RP-HPLC reaction-product readout, no modification to the method was necessary in order to observe the reaction products generated using the SYNTAXtide^®^560 substrate. 

Trypsin (TrypZean, Sigma-Aldrich Corp.; St. Louis, MO, USA) was utilized to identify characteristic trypsin cleavage while Serotype C light-chain (List Biological Laboratories, Inc.; Campbell, CA, USA) was obtained for type-C cleavage generation.

### 2.1. Enzymatic (Light-chain) Reaction Conditions

Each BoNT/A product vial was reconstituted with 1 mL of digestion buffer (see Table 2). 

**Table 2:** Reaction solutions.

**Digestion buffer**	0.5 mM ZnCl_2_, 2 mM DTT ^a^, 0.05% Tween 20 in 50 mM HEPES, pH 7.4
**Substrate**	200 µM SNAPtide^®^520 ^b^ prepared in sterile water for injection

^a ^DTT = Dithiothreitol. ^b ^List Biological Laboratories, Inc., supplied through Calbiochem, San Diego, CA, USA (Cat #567333).

A 0.45 mL aliquot of each reconstituted sample was transferred to separate reaction tubes. The samples were then heated at 37 °C for 30 minutes (reduction step). After completion of the reduction step, 25 µL of 200 µM SNAPtide^®^520 was then added to each tube (equivalent to 10.5 µM SNAPtide^®^520). For the experiment using the SYNTAXtide^®^560 substrate (type-C), a volume of 50 µL of 200 µM SYNTAXtide^®^560 was added to each reaction tube (equivalent to 20 µM SYNTAXtide^®^ 560).

The reaction samples were then incubated at 30 °C for 20 hours (digestion step). At the completion of the digestion step, 25 µL of 5% trifluoroacetic acid (TFA) was added to each tube to stop the reaction. The contents of each tube were then transferred to HPLC vials for analysis.

### 2.2. HPLC Analysis

The fluorescently labeled cleavage product(s) were separated and detected via an RP-HPLC method using a Waters 2695 XE Separations Module and a Waters 2475 Multi λ Fluorescence Detector (see Table 3 for HPLC parameters). 

**Table 3:** HPLC parameters.

**Column**	Waters Symmetry300™ C18, 3.5 µm, 4.6 × 150 mm (P/N: 186000197)
**Column Temperature**	35 °C
**Injection Volume**	25 µL
**Flow**	1 mL/min
**Detection**	Excitation λ = 322 nm; Emission λ = 420 nm

The samples were eluted using a gradient program (see Table 4) with a mobile phase consisting of 0.1% TFA in water (A) and 0.1% TFA in acetonitrile (B). The data were collected and analyzed via Waters Empower™ Pro software (Waters Corp.; Milford, MA, USA).

**Table 4:** HPLC Gradient Program.

**Time (min)**	**%A**	**%B**
0	90	10
5	90	10
13	85	15
18	5	95
20	5	95
21	90	10
30	90	10

## 3. Results and Discussion

**Figure 1:**
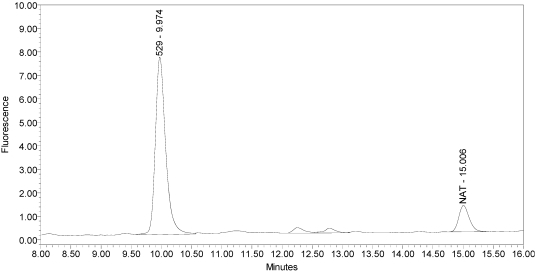
Chromatogram of onabotulinumtoxinA (BOTOX^®^ Lot #C1550C1) reaction solution (SNAPtide^®^520 substrate) exemplifying generation of the 529 fragment peak.

The LCA-HPLC method demonstrated excellent single-vial sensitivity and resolution of the fluorescently labeled BoNT/A cleavage fragment (designated as SNAPtide^®^529 by List Biological Laboratories, Inc.) when analyzing onabotulinumtoxinA, abotulinumtoxinA and incobotulinumtoxinA drug products. See Figure 1 for an example chromatogram of onabotulinumtoxinA. No formulation or reaction component interferences were observed. 

An excipient peak, identified as *N*-Acetyl-tryptophan (NAT), exists in all the commercial product chromatograms but is readily resolved from the reaction products. NAT is used as a stabilizer in human serum albumin (HSA). OnabotulinumtoxinA, abotulinumtoxinA, and incobotulinumtoxinA drug products all contain HSA. 

**Figure 2:**
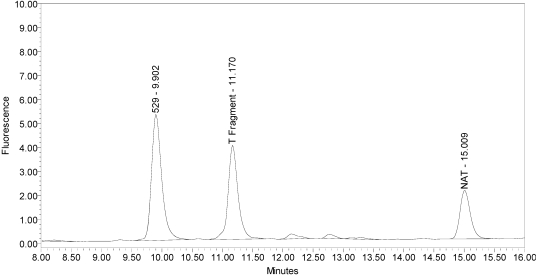
Chromatogram of incobotulinumtoxinA (Xeomin^®^ Lot #408012) reaction solution (SNAPtide^®^520 Substrate) exemplifying generation of the 529 fragment peak and the atypical “T fragment” peak.

All BoNT/A drug products tested generated the expected BoNT/A substrate cleavage product. However, when analyzed using the LCA-HPLC method, the incobotulinumtoxinA drug product generated two (2) substrate cleavage products (see Figure 2). 

**Figure 3:**
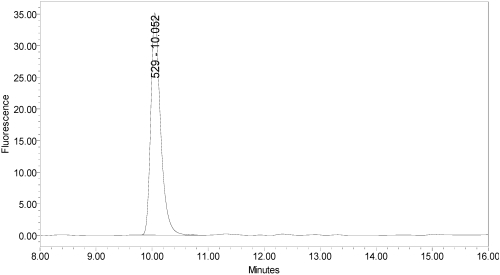
Chromatogram of abotulinumtoxinA (Dysport^®^ Lot #589AB) reaction solution (SNAPtide^®^520 Substrate) exemplifying generation of the 529 fragment peak.

Under identical reaction conditions, onabotulinumtoxinA generated only the expected BoNT/A substrate cleavage product (see Figure 1), as did abotulinumtoxinA (see Figure 3). 

**Figure 4:**
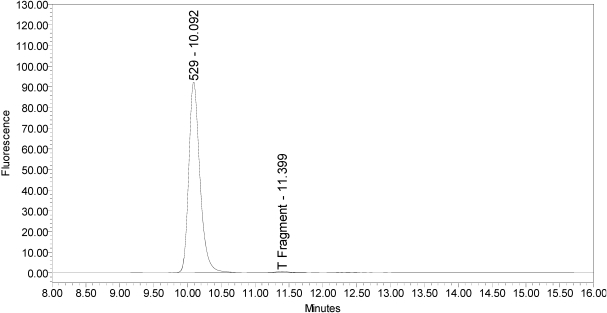
Chromatogram of 150-kD BoNT/A reaction solution (SNAPtide^®^520 Substrate) exemplifying generation of the 529 fragment peak.

**Figure 5:**
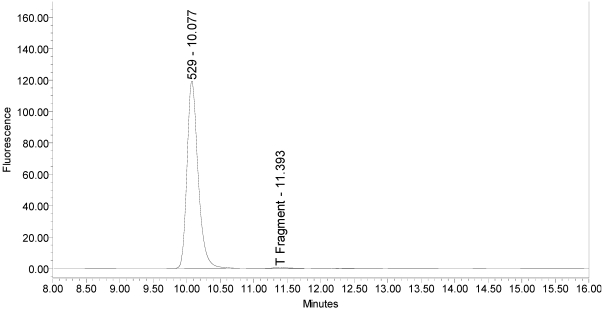
Chromatogram of 900-kD BoNT/A reaction solution (SNAPtide^®^520 substrate) exemplifying generation of the 529 fragment peak.

In addition, samples of bulk 150-kD BoNT/A and 900-kD BoNT/A only generated the single expected BoNT/A substrate cleavage product [7](see Figure 4 and Figure 5).

**Figure 6:**
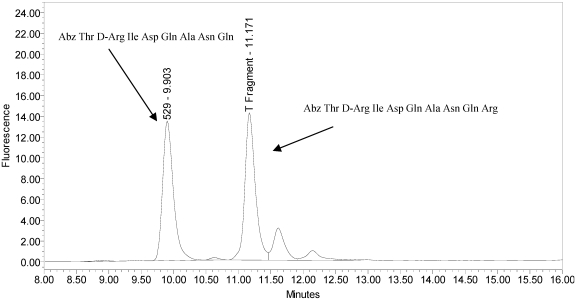
Chromatogram of synthesized samples of SNAPtide^®^529 and “T fragment” for retention time identification. Elution positions match the generated cleavage fragments.

**Figure 7:**
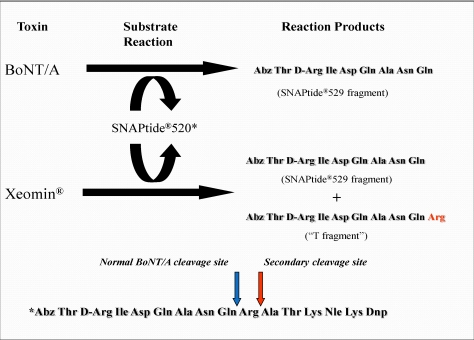
BoNT/A Reaction Scheme with SNAPtide^®^520 Substrate.

The secondary cleavage product observed from incobotulinumtoxinA was identified by HPLC retention time as the SNAPtide^®^529 fragment plus one amino acid (see Figure 6 and Figure 7). 

This was accomplished by injecting a range of synthesized SNAPtide^®^520 fragments along with the SNAPtide^®^529 fragment and comparing the retention times. The sequence for the SNAPtide^®^520 substrate is given in the List Biological Laboratories, Inc., patent [8]. BoNT/A specifically cleaves SNAP-25_1–206_ at position Gln197–Arg198, while the secondary cleavage product is the result of cleavage of SNAP-25_1–206_ at Arg198–Ala199. Trypsin also cleaves at Arg198–Ala199 (data on file); hence, this secondary cleavage product observed from incobotulinumtoxinA was termed the “T fragment” for convenience. It should be noted that this is also the cleavage site for serotype C toxin (BoNT/C)[9].

**Figure 8:**
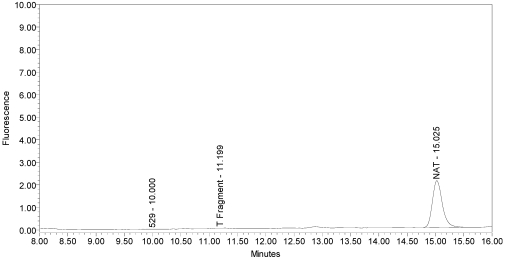
Chromatogram of incobotulinumtoxinA (Xeomin^®^ Lot #408012) reaction solution without the addition of substrate demonstrating drug product generates no peaks in the absence of substrate.

A control reaction was run on the incobotulinumtoxinA product, without the SNAPtide^®^520 substrate, to elucidate whether the observed peak was indeed due to the cleavage of the substrate or whether it was an extraneous peak due to the formulation ingredients or to a formulation matrix interaction with a reaction component (see Figure 8). 

No peak eluting at the retention time of the “T fragment” peak was observed in this control reaction, confirming that the “T fragment” peak generated by incobotulinumtoxinA was indeed due to enzymatic cleavage of SNAPtide^®^520 substrate.

**Figure 9:**
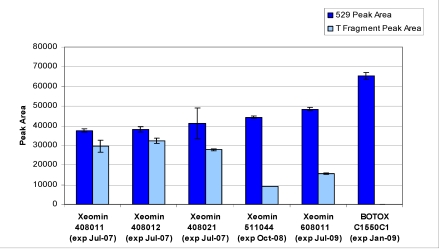
Peak area results for incobotulinumtoxinA (Xeomin^®^) samples compared to onabotulinumtoxinA (BOTOX^®^) control. Samples analyzed by LCA-HPLC using the SNAPtide^®^520 substrate. Test Date: April 2007, all samples tested within expiry.

Peak area results for five incobotulinumtoxinA drug product lots along with a onabotulinumtoxinA drug product lot are given in Figure 9. All incobotulinumtoxinA drug products assayed demonstrated significant levels of secondary cleavage. 

**Figure 10:**
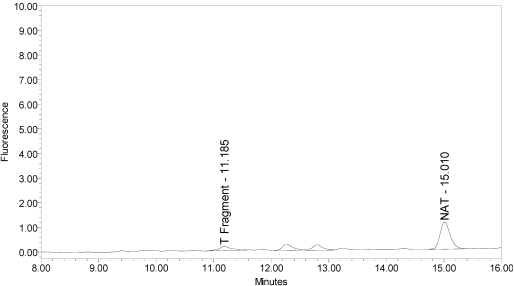
Chromatogram of onabotulinumtoxinA (BOTOX^®^ Lot #C1550C1) reaction solution without the reduction step (no DTT added) demonstrating the 529 peak is not generated from by onabotulinumtoxinA from the SNAPtide^®^520 substrate without reduction.

When onabotulinumtoxinA drug product was analyzed without reduction, *i.e.*, DTT omitted from the reaction buffer, no substantial reaction products were observed (see Figure 10). 

**Figure 11:**
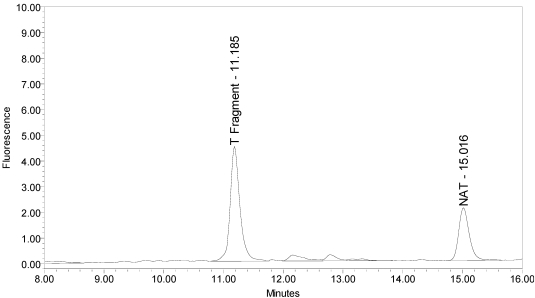
Chromatogram of incobotulinumtoxinA (Xeomin^®^ Lot #408012) reaction solution without the reduction step (no DTT added) exemplifying the “T fragment” peak is generated by incobotulinumtoxinA from the SNAPtide^®^520 substrate without reduction.

However, when incobotulinumtoxinA drug product was analyzed without the reduction step (no DTT), the secondary cleavage product (“T fragment”) was still generated (see Figure 11), indicating reduction is not necessary for this cleavage. No SNAPtide^®^529 fragment (primary BoNT/A cleavage product) was detected when testing incobotulinumtoxinA drug product under non-reduced conditions.

**Figure 12:**
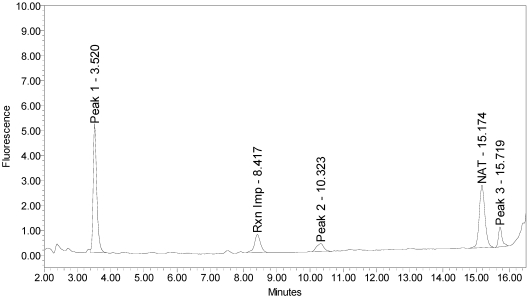
Chromatogram of incobotulinumtoxinA (Xeomin® Lot #708041) reaction solution with SYNTAXtide^®^560 substrate (serotype C substrate) demonstrating the incobotulinumtoxinA BoNT/A drug product cleaves serotype C substrate.

When reacted with the SYNTAXtide^®^560 substrate (serotype C substrate), incobotulinumtoxinA drug product generated a major peak eluting at 3.5 minutes along with two (2) small peaks eluting at 10.3 and 15.7 minutes, respectively (see Figure 12). 

**Figure 13:**
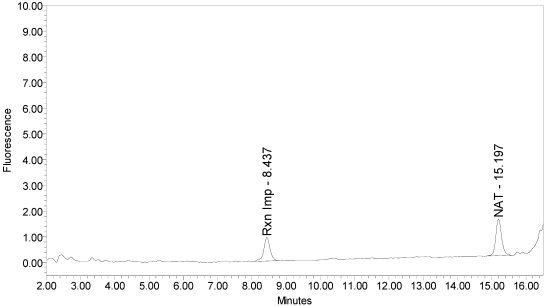
Chromatogram of onabotulinumtoxinA (BOTOX® Lot #408012) reaction solution with SYNTAXtide^®^560 substrate (serotype C substrate) exemplifying the onabotulinumtoxinA BoNT/A drug product does not cleave serotype C substrate.

**Figure 14:**
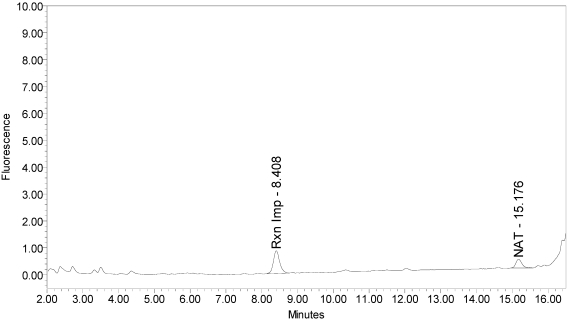
Chromatogram of abotulinumtoxinA (Dysport^®^ Lot #710A) reaction solution with SYNTAXtide^®^560 substrate (serotype C substrate) exemplifying the abotulinumtoxinA BoNT/A drug product does not cleave serotype C substrate.

Neither onabotulinumtoxinA or abotulinumtoxinA generated any cleavage products when reacted with the SYNTAXtide^®^560 substrate (see Figure 13 and Figure 14). 

**Figure 15:**
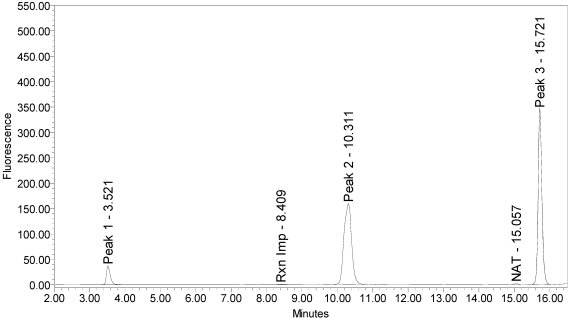
Chromatogram of trypsin reaction solution with SYNTAXtide^®^560 substrate (serotype C substrate) demonstrating trypsin cleavage pattern for serotype C substrate.

Trypsin also produced three (3) reaction products that corresponded to the retention times of the incobotulinumtoxinA reaction products, however at different ratios (see Figure 15). 

**Figure 16:**
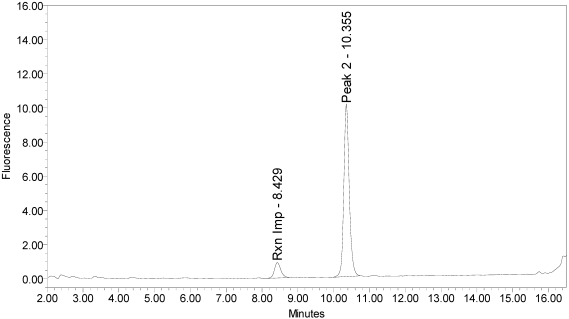
Chromatogram of serotype C light-chain reaction solution with SYNTAXtide^®^560 substrate (serotype C substrate) demonstrating typical serotype C cleavage pattern against serotype C substrate.

For trypsin, the peaks eluting at 10.3 and 15.7 minutes were the largest, with a smaller peak at eluting 3.5 minutes. Serotype C light-chain generated a single peak eluting at 10.4 minutes (see Figure 16). 

A summary of the cleavage patterns observed using the SYNTAXtide^®^560 substrate is given in Table 5. It should be noted that the peak eluting at 8.5 minutes observed in all the chromatograms (labeled as reaction impurity) is from an impurity in one of the reaction components.

**Table 5:** Summary of cleavage products observed with SYNTAXtide^®^560 substrate (serotype C substrate) for drug products and control enzymes.

	**Retention Time**		
	**3.5 minutes**	**10.3 minutes**	**15.7 minutes**
onabotulinumtoxinA	Non-detected	Non-detected	Non-detected
abotulinumtoxinA	Non-detected	Non-detected	Non-detected
incobotulinumtoxinA	Major peak	Minor peak	Minor peak
Serotype C light-chain	Non-detected	Major peak	Non-detected
Trypsin	Minor peak	Major peak	Major peak

## 4. Conclusions

The suitability of the method was exemplified through analysis of three distinct BoNT/A drug products. The method was shown to be robust and sensitive as applied to single vials of drug products with very different formulations. Light-chain activity ranked abotulinumtoxinA > onabotulinumtoxinA > incobotulinumtoxinA. It should be noted that the abotulinumtoxinA product label stated potency as 500 U, so it was not surprising that it produced more enzymatic activity, whereas the incobotulinumtoxinA label claims 100 U, although it consistently assayed significantly lower than the other 100 U product (onabotulinumtoxinA). 

IncobotulinumtoxinA drug product generated a secondary SNAP cleavage product not produced at significant levels by any other BoNT/A drug product or BoNT/A bulk toxin tested. Possible explanations include damaged BoNT/A or a contaminating protease that cleaves SNAPtide^®^520 substrate at the *C*-terminal end of arginine (corresponding to SNAP-25 Arg198–Ala199). Based on the cleavage site, suspected candidates for a contaminating protease could include endogenous cell-derived trypsin-like protease or toxin co-expression during fermentation (serotype C and A behavior). We eliminated co-expression of serotype C as a plausible explanation because while serotype C light-chain cleaves SYNTAXtide^®^560 it does not cleave SNAPtide^®^520 (supplier information verified by data on file) and therefore would not produce the T fragment. Another explanation is that the type-A holotoxin is damaged during processing and/or degraded during storage, thereby altering its proteolytic behavior [10]. To our knowledge, this is the first reported observation of this unusual behavior related to a BoNT/A toxin. In addition, older lots of incobotulinumtoxinA consistently exhibited lower light-chain activity (type-A cleavage) when compared to newer lots, combined with an increase in the ratio of secondary substrate cleavage product to primary cleavage product (see Figure 9); suggesting the incobotulinumtoxinA drug product may be degrading during storage.

While the origin and nature of the secondary substrate cleavage produced by incobotulinumtoxinA is unknown, a difference in the proteolytic mechanism was elucidated by this study. This difference was observed when drug product samples were analyzed without reduction with DTT. In order for BoNT/A light-chain activity to occur, the disulfide link between the 100-kD heavy-chain and the 50-kD light-chain must be reduced. As expected, no BoNT/A cleavage product (SNAPtide^®^529) was observed without reduction in any samples. However incobotulinumtoxinA samples generated the secondary fragment in the absence of a reduction step, consistent with a different proteolytic mechanism. In any event it seems evident that some enzymatic activity is directed to the secondary mechanism and may also be contributing to loss during storage; e.g., a degrative contaminant protease.

In an expanded experiment, incobotulinumtoxinA demonstrated cleavage of a BoNT/C substrate that was not observed in the other BoNT/A drug products tested. The cleavage pattern of the BoNT/C substrate by incobotulinumtoxinA was inconsistent with that of trypsin and BoNT/C light-chain. It is interesting to note that this is the only drug product utilizing the naked 150-kD holotoxin. A comprehensive understanding of BoNT/A drug product differentiation would benefit from additional investigation into this phenomenon now that a tool exists to detect and measure it.
